# Supplementary material for: Product progression: a machine learning approach to forecasting industrial upgrading
Source: Sci Rep. 2023 Jan 27;13:1481. doi: 10.1038/s41598-023-28179-x (PMC9880377; doi:10.1038/s41598-023-28179-x)
Supplement: Supplementary file 1 — Supplementary Information. [file 41598_2023_28179_MOESM1_ESM.pdf]

Supplementary Information
Product Progression - A machine learning approach to forecasting industrial upgrading

Giambattista Albora, Luciano Pietronero, Andrea Tacchella, Andrea Zaccaria

1 Tuning of the hyperparameters

In this section we discuss the tuning of the hyperparameters of the different machine learning models used in the text. The procedure we adopt is to train the models using different hyperparameters on the data from 1996 to 2008 and then to to validate the results on the data relative to the year 2013. In this way, we find the best values for the models' hyperparameters and then we use these values to train the models as described in the method section (that is, using 1996-2013 data and/or cross-validation). To evaluate the performance of the models on the validation set we use the Area Under the Precision Recall Curve (AUC-PR); this is a common procedure to take into account both false positives and false negatives without having to choice an arbitrary threshold. For more details about the models and their hyperparameters we refer to the relevant literature [3].

1.1 Random Forest

When training a Random Forest, two important hyperparameters are *min_ samples_ leaf* and *n_ estimators* [5]. min_samples_leaf imposes the minimum number of samples that a leaf node can hold. The default value of the python library we are using, sklearn, is 1, which means that each single tree of the Random Forest is built in order to perfectly classify the training data. This could lead to overfitting, so increasing the value of min_samples_leaf could improve the results. However if the value is too high, it may happen that we are limiting too much the trees, so the performance of the Random Forest decreases. From the tuning of min_samples_leaf we obtain the curve in figure 1 on the left, so min_samples_leaf = 7 is the optimal trade-off between avoiding overfitting and not limiting the trees too much.
n_estimators is the number of trees that compose the Random Forest. If it is too low we may not have enough statistics to perform a good classification, while the disadvantage of using too much trees is the increase of the computational time needed to train the model. From the tuning of n_estimators (with min_samples_leaf = 7) we get the curve in figure 1 on the right. We conclude that n_estimators = 100 is a value for which we have enough statistics while keeping a good computational performance, since increasing the number of trees does not significantly improve the performance.

1.2 ExtraTreesClassifier

To optimize the ExtraTrees Classifier, we follow the same procedure we applied to the Random Forest. In figure 2 we show the results of the tuning of min_samples_leaf and n_estimators. The chosen values are min_samples_leaf = 8 and n_estimators = 100.

1.3 XGBoost

When we talk about XGBoost [2] the n_estimators hyperparameter has a different behavior with respect to its equivalent of the Random Forest, indeed XGBoost is a boosting algorithm and using too much trees can lead to overfitting and reduce the performance. In addition to n_estimators we took into consideration also the min_child_weight and reg_lambda hyperparameters. The former has a similar behavior of min_samples_leaf and the latter is a L2 regularization term. We apply a grid search with the following values:

- n_estimators: 5, 10, 15, 30, 60

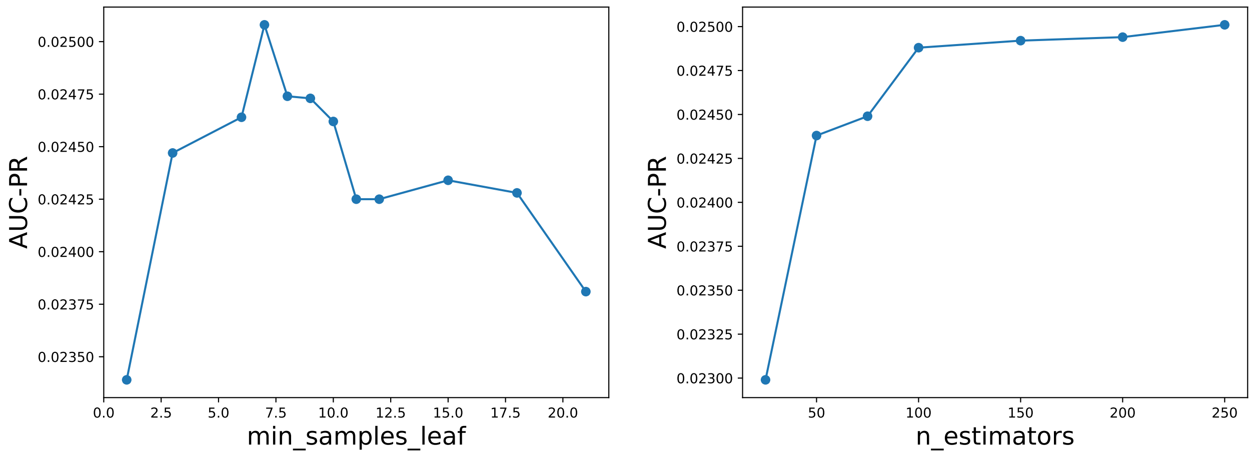

Figure 1: Tuning of the min_samples_leaf (left) and n_estimators (right) hyperparameters of the Random Forest. The chosen values are min_samples_leaf = 7 and n_estimators = 100.

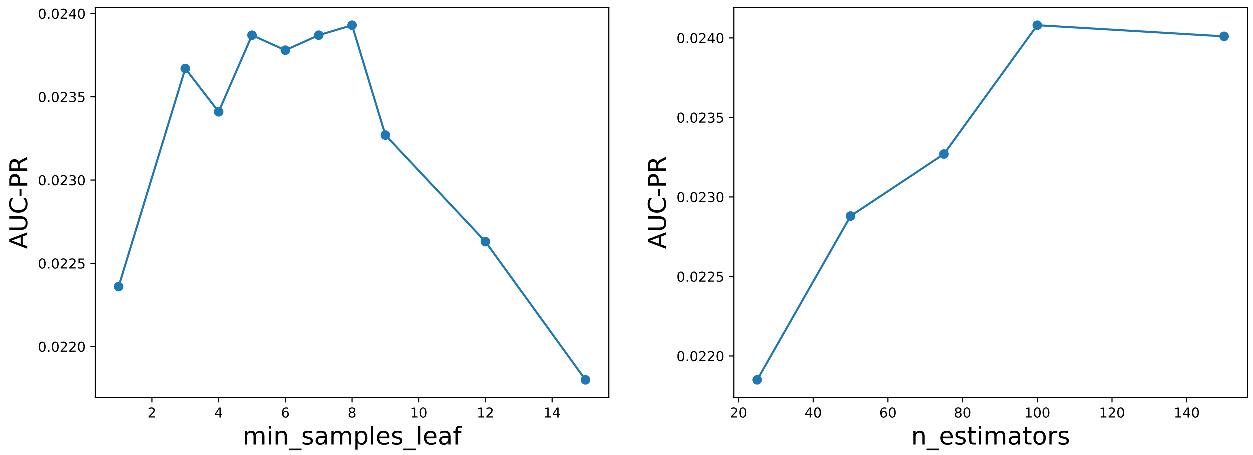

Figure 2: Tuning of the min_samples_leaf (left) and n_estimators (right) hyperparameters of the ExtraTrees Classifier. The chosen values are min_samples_leaf = 8 and n_estimators = 100.

- min_child_weight: 5, 15, 30, 45, 60
- reg_lambda: 1.0, 1.25, 1.5, 1.75, 2.0

Due to the XGBoost high computational time and the number of training a grid search requires, for the this optimization we considered only 100 products.
The optimal configuration turned out to be n_estimators = 15, min_child_weight = 45 and reg_lambda = 1.5.

1.4 ADABoost

ADABoost is an algorithm that starts with a base estimator and then apply a boosting procedure. In our case, we use the default estimator in the sklearn implementation of ADABoost consisting into a decision tree with depth = 1. In figure 3 there is the tuning of the n_estimators hyperparameter. The chosen value is n_estimators=3

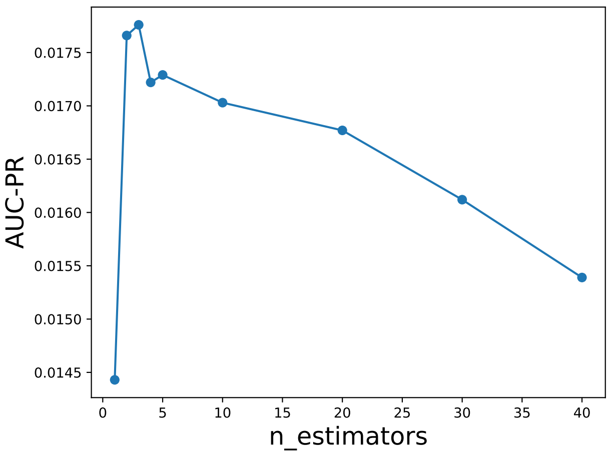

Figure 3: Tuning of the n_estimators hyperparameter of ADABoost. The chosen value is n_estimators = 3.

1.5 Support Vector Machine

The Support Vector Machine is a kernel-based method and, as such, its most important hypeparameter is the choice of the kernel function [4]. For a binary classification, the sklearn library proposes four different kernels: linear, poly (polynomial), rbf (radial basin function) and sigmoid. In figure 4 we show the performance of each of these kernels and the conclusion is that rbf is the best choice.

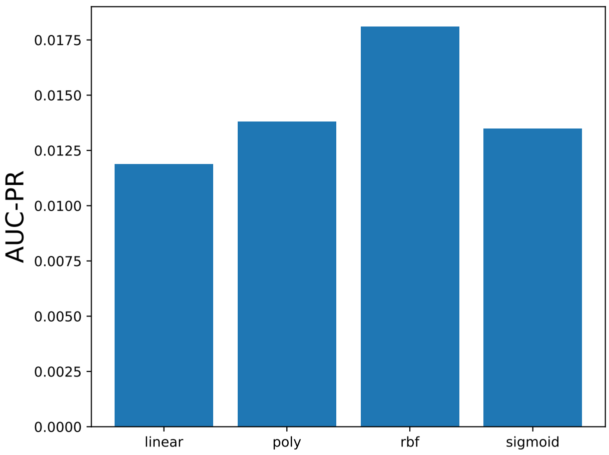

Figure 4: Performance of different kernels for the Support Vector Machine. The optimal one is rbf.

1.6 Dense Neural Network

Our dense neural network consists into 2 hidden layers with 32 neurons and activation function RELU and a final layer with a single neuron and sigmoid activation function. We used the Keras library, and in particular rmsprop (Root Mean Squared Propagation) as optimizer, binary_crossentropy as loss function, and accuracy as loss metric. In figure 5 on the left we try different values for the number of neurons of the first 2 layers finding that the optimal one is 64. On the right we compare different optimizers implemented in keras finding that the best one is RMSprop. Finally in figure 6 we show the accuracy of the classification during the training and validation for three neural networks that predict the future export of three different products. As the reader can see using more than 10 epochs does not bring to an improvement of the accuracy in the validation set.

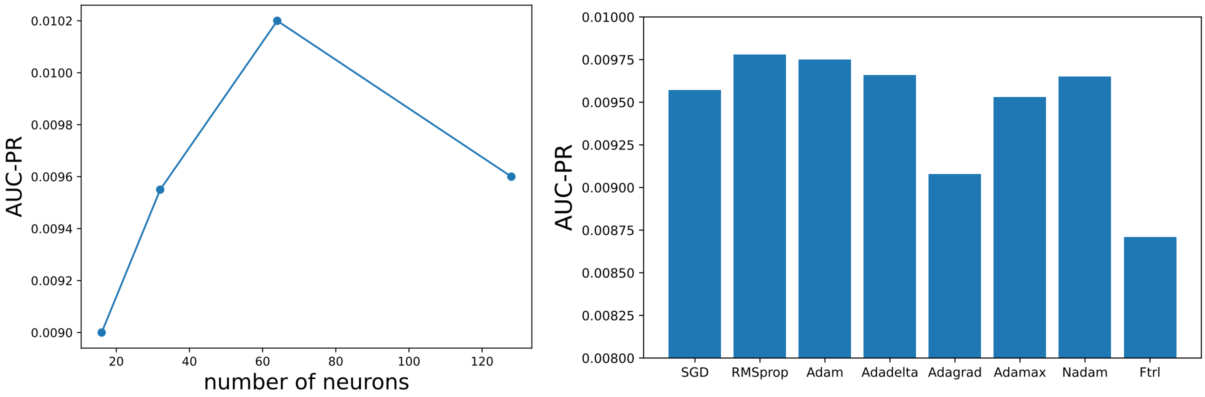

Figure 5: Prediction performance on the validation set using a dense neural network and using different numbers of neurons in the hidden layers (on the left) and different optimizers (on the right). The optimal number of neurons is 64 while the best optimizer is RMSprop.

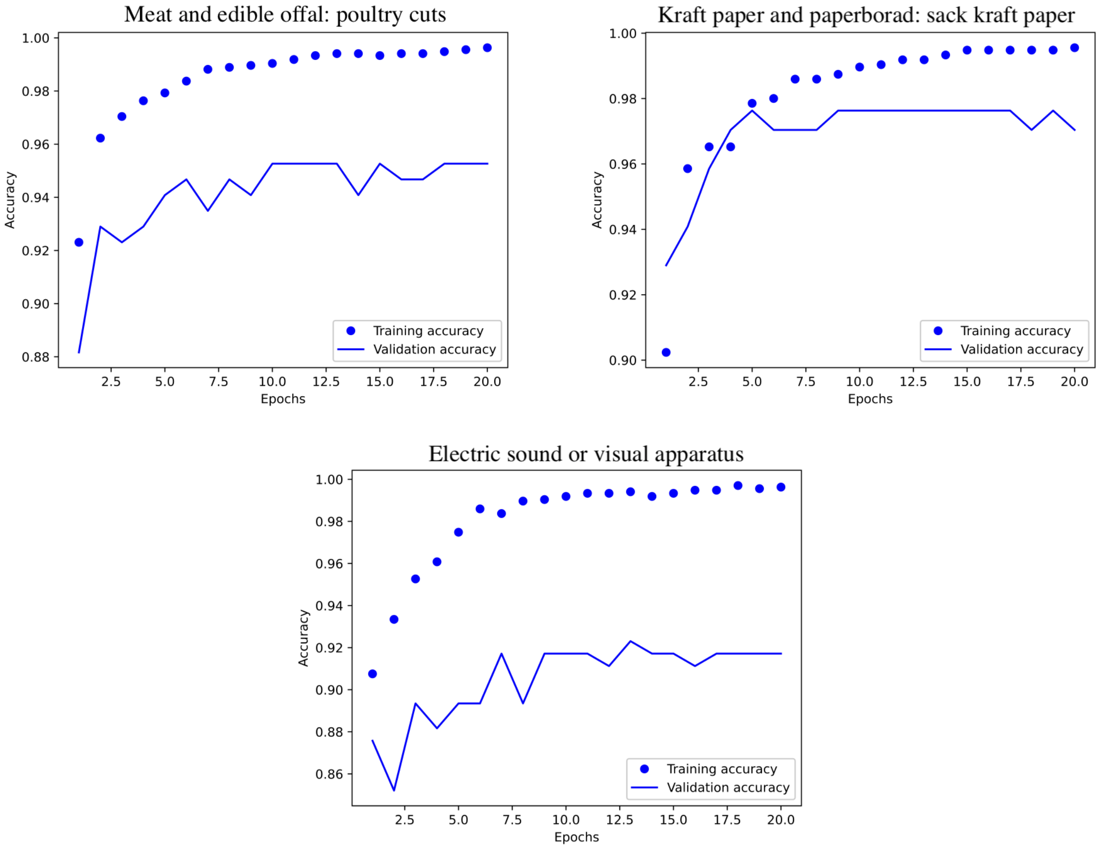

Figure 6: Accuracy of the neural network during the training and the validation. After 10 epochs in the validation curve there is no improvement of the accuracy.

2 Training considering the class imbalance

Since our dataset is heavily class imbalanced, one may argue whether this affects our results and how to take it into account. A classic way to try fixing the class imbalance issue is to build the training set by extracting subsamples in which one gives different weights to the positive and negative instances [3]. In particular, the

weight of the positives is $w = \frac{\#\ of\ negatives}{\#\ of\ positives}$. This can be accomplished by setting the value class_weight = "balanced" in the sklearn library models, while in XGBoost the weight of the positive elements can be set by using the scale_pos_weight parameter. In table 1 we show the AUC-PR of our models with and without this change of weights. Quite surprisingly, the performance of the models decreases. In doing this comparison we consider Random Forest, ExtraTreesClassifier and XGBoost both trained using the respective default values of min_samples_leaf and min_child_weight and using the values we find with the optimization described in the previous section. Here we include also a different implementation of the Random Forest that is the Balanced Random Forest Classifier of the imblearn python library.

**Algorithm**	**same weights**	**class_weight = "balanced"**
BalancedRandomForest	0.0176	0.0133
Random Forest	0.0234	0.0200
Random Forest min_samples_leaf=7	0.0251	0.0224
ExtraTreesClassifier	0.0224	0.0192
ExtraTreesClassifier min_samples_leaf=8	0.0239	0.0227
XGBoost	0.0199	0.0174
XGBoost min_child_weight=30	0.0231	0.0178
Logistic Regression	0.0104	0.0101
Support Vector Machine	0.0181	0.0178

Table 1: AUC-PR of the models before and after modifying the weight of the positive instances in order to compensate their minority.

Finally, we also tried to train the Random Forest generating new positive instances in the training set with the use of the SMOTE procedure [1]. The AUC-PR of the Random Forest is 0.0234, while the AUC-PR of the Random Forest with SMOTE is 0.0203. So also the use of SMOTE decreases the performance.

3 Cross Validation

Here we show how the performance of the three most performing algorithms (the tree-based algorithms Random Forest, ExtraTrees Classifier, and XGBoost) changes when we perform the 13-fold cross validation. In figure 7 we show that while Random Forest and ExtraTrees get worse, XGBoost gets better. We argue that the different behavior of the algorithms is due to the fact that without cross-validation XGBoost focus on the high autocorrelation of the export data, so removing the country on which we make the predictions from the training set forces XGBoost to learn the complex interdependencies among products. Random Forest and ExtraTreesClassifier are less influenced from the high autocorrelation of the data, so they do not need a cross-validation.

4 Evaluation metrics and AUC_PR for parameters optimization

In this work we compare different metrics to evaluate the goodness of the forecasts. Some of these metrics depend on the threshold defining positive and negative predictions. As a reference value for this threshold we take the one that maximizes the F1 score.
Being the F1 score an harmonic mean of precision and recall, its maximum value does not always correspond to both a high precision and a high recall. To make a practical example, the non cross-validated Random Forest has an higher F1-score than the cross-validated XGBoost, however the latter has an higher recall than the former (see table table 1 of the main text).
The use of F1 scores permits to balance the need to have a relatively low number of both false positives and false negatives, however, the weak point is its dependence on the threshold, and for this reason one could prefer indicators such as the AUC_ROC, AUC_PR and mean Precision@10. However, the AUC_ROC suffers from the high class imbalance of the data, and the mean Precision@10, despite having a direct and intuitive meaning, fluctuates a lot, so it is not indicated to make a tuning of the hyperparameters. In figure 8 on the left we report the tuning of the min_samples_leaf of the Random Forest using mean Precision@10, as one can see the fluctuations of this metric make it difficult to find a clear peak. In the same figure on the right we use Best F1. Even if there is a peak corresponding to min_samples_leaf = 7 like in figure 1, the Best F1 values fluctuates

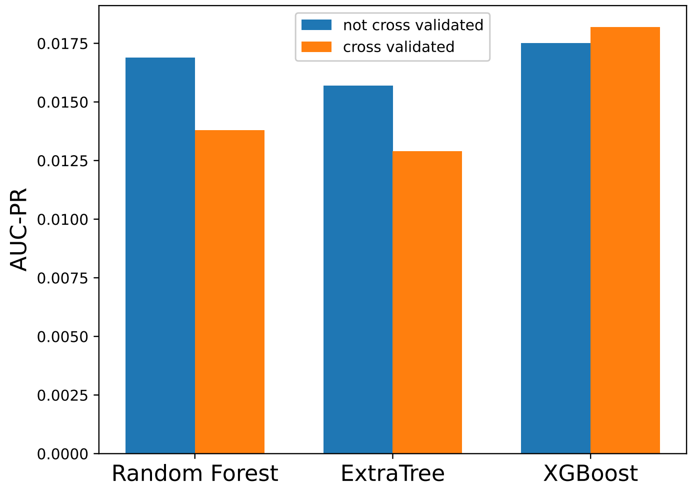

Figure 7: Performance of Random Forest, ExtraTrees and XGBoost with and without the cross validation. While Random Forest and ExtraTrees get worse, XGBoost gets better.

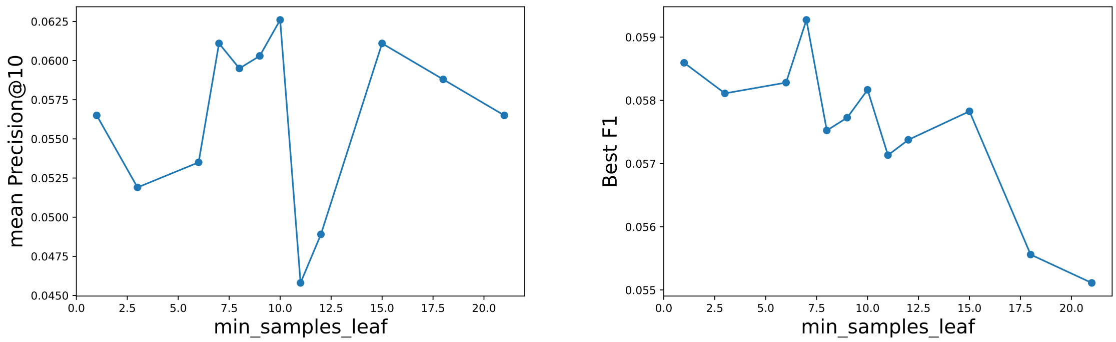

Figure 8: Tuning of the min_samples_leaf hyperparameter of the Random Forest using mean Precision@10 (on the left) and Best F1 (on the right).

more with respect to AUC-PR, and for this reason we use the latter for the tuning of the hyperparameters. In conclusion, the AUC_PR represents the best choice to make a tuning of the hyperparameters, since it is stable, it does not depend on any arbitrary threshold, and it is not affected by the high class imbalance since it does not consider the True Negatives.

References

[1] Nitesh V Chawla, Kevin W Bowyer, Lawrence O Hall, and W Philip Kegelmeyer. Smote: synthetic minority over-sampling technique. *Journal of artificial intelligence research*, 16:321–357, 2002.

[2] Tianqi Chen and Carlos Guestrin. Xgboost: A scalable tree boosting system. In *Proceedings of the 22nd acm sigkdd international conference on knowledge discovery and data mining*, pages 785–794, 2016.

[3] Aurélien Géron. *Hands-on machine learning with Scikit-Learn, Keras, and TensorFlow: Concepts, tools, and techniques to build intelligent systems.* " O'Reilly Media, Inc.", 2019.

[4] Patrick Koch, Bernd Bischl, Oliver Flasch, Thomas Bartz-Beielstein, Claus Weihs, and Wolfgang Konen. Tuning and evolution of support vector kernels. *Evolutionary Intelligence*, 5(3):153–170, 2012.

[5] Philipp Probst, Marvin N Wright, and Anne-Laure Boulesteix. Hyperparameters and tuning strategies for random forest. *Wiley Interdisciplinary Reviews: data mining and knowledge discovery*, 9(3):e1301, 2019.
